# Anticipatory and Anticipated Emotions in Regular and Non-regular Exercisers – A Qualitative Study

**DOI:** 10.3389/fpsyg.2022.929380

**Published:** 2022-07-04

**Authors:** Katharina Feil, Susanne Weyland, Julian Fritsch, Hagen Wäsche, Darko Jekauc

**Affiliations:** Institute of Sports and Sports Science, Karlsruhe Institute of Technology, Karlsruhe, Germany

**Keywords:** anticipatory, anticipated, emotion, affect, physical activity, exercising

## Abstract

Future-oriented emotions could influence our decisions in everyday life and help understand why some individuals are physically active whilst others are not. Current literature distinguishes between two future-oriented emotion constructs: anticipatory and anticipated emotions. While anticipatory emotions are currently experienced emotions about a future event, anticipated emotions refer to the emotions that a person is expected to experience when confronted with a future event. The main aims of the present study were (1) to identify and describe (a) categories of anticipatory emotions experienced before exercise, and (b) categories of anticipated emotions expected to be experienced during and after exercise, and (2) to develop a theoretical model of anticipated emotion categories. Sixteen participants (*M*_*age*_ = 26.03, *SD* = 6.66) were recruited for semi-structured interviews, and their statements were analyzed using principles of the Grounded Theory. In total, 13 different anticipatory and anticipated emotion categories were identified, such as enjoyment, anxiety, pride, self-anger, and relief. Anticipatory emotions seem to reflect the current affective valence of exercising and may be influenced by daily factors. With regards to anticipated emotions, the results show that regular exercisers anticipated also negative emotions such as anxiety, disappointment, and self-anger, and non-regular exercisers also anticipated positive emotions such as enjoyment, pride, and satisfaction. Therefore, future research should not only focus on the valence of future-oriented emotions, but should investigate the possible impact of specific anticipated emotions on exercise behavior. In addition, a theoretical model of anticipated emotion categories in exercise behavior derived from the interviews. The model outlines different categories of anticipated emotions based on appraisal processes. In conclusion, we assume that this developmental process of anticipated emotions may be embedded in a broader, cyclical process within the context of exercising.

## Introduction

Even though the benefits of regular exercise behavior on physical (Reiner et al., 2013) and mental health (White et al., 2017) are widely known, studies show that across countries, people are not sufficiently active (Hallal et al., 2012; Marques et al., 2015). Worldwide, 31.1% of adults are physically inactive and do not meet the physical activity guidelines (Hallal et al., 2012). In a European study, participants older than 24 were more likely to attain the recommended physical activity guidelines compared to 18–24-year-old ones (Marques et al., 2015). Therefore, young adults seem to be a vulnerable group when it comes to physical activity promotion. Considering the relevance of physical activity, it is not surprising that there is a great interest in understanding the psychological processes that can influence an individual’s decision about being physically active or not. According to the *pleasure principle*, individuals are drawn to behaviors associated with pleasure and avoid behaviors associated with displeasure (Higgins, 1997). Behaviors are associated with a certain level of affect and these associations influence the decision whether or not to carry out a behavior (Slovic et al., 2007). Future-oriented emotions could play a crucial role in the decision-making process regarding the execution of physical activity. The theoretical approach of this article focuses on the anticipation of emotions related to future physical activity behavior rather than looking at currently experienced emotions during physical activity (Baumeister et al., 2007; DeWall et al., 2015).

In exercise psychology, the concept of affect has become the focus of recent research. Affect is an umbrella term for phenomena related to moods and emotions (Russell, 2003; Barrett, 2006). In particular, the term *core affect* is used to describe a “neurophysiological state consciously accessible as the simplest raw (non-reflective) feeling evident in moods and emotions” (Russell, 2003, p. 148). In the circumplex model (Russell, 2003), *affective states* are located on two dimensions that characterize core affect: valence (pleasure – displeasure) and arousal (deactivated – activated). Emotions are context-dependent and more complex than core affect (Russell, 2003; Barrett, 2006; Clore and Ortony, 2013). According to Ortony et al. (1988, p. 13), emotions are “represented as a set of substantially independent groups based on the nature of their cognitive origins.” In terms of future-oriented emotions, anticipatory emotions and anticipated emotions are considered two central constructs (Baumgartner et al., 2008). On the one hand, *anticipatory emotions* are currently experienced emotions about a future event (Bagozzi et al., 1998; Baumgartner et al., 2008). For instance, a person experiences the emotion anxiety in the moment they think about the next exercise session. On the other hand, *anticipated emotions* refer to the emotions that a person is expected to experience when confronted with a future event (Perugini and Bagozzi, 2001; Baumgartner et al., 2008). For example, in an imaginary prediction, a person expects to experience enjoyment during the next exercise session. Expectancies are defined as “beliefs about a future state of affairs” based on past experiences (Roese and Sherman, 2013, p. 91). Thus, the anticipated emotions can be considered a cognitive construction of a future state based on expectancies. It is assumed that they are either consciously or unconsciously stored in form of mental models which can be understood as internal representations of the world constructed by the individual (Jones et al., 2011; Saban, 2019).

Focusing on anticipatory emotions, Bagozzi et al. (1998) were looking for prospective emotions that may influence motivation and goal-achievement regarding their body weight (success or failure). They showed that positive anticipatory emotions were a significant predictor of motivation and future health behaviors, such as exercising and dieting (Bagozzi et al., 1998). However, it should be mentioned that items to assess anticipatory emotions were rather formulated as a measurement of anticipated emotions (“If I succeed/do not succeed to … I will feel …”). In an attempt to distinguish more between these two constructs, Baumgartner et al. (2008) studied anticipatory and anticipated emotions independently. The study was about the millennium change and its possible negative impact on the country and on the participants’ personal lives. Regarding anticipatory emotions, participants rated how worried, anxious, uncomfortable, optimistic, and confident they currently felt about the millennium change and the consequences during the first days of 2000. Anticipated emotions regarded the imagined emotions during the first week of 2000. The results indicated that both anticipatory and anticipated emotions independently influenced behavior intentions to limit possible negative consequences caused by the millennium change. Moreover, the study showed that anticipatory emotions were mainly reflected in the prospective emotions hope and fear, while anticipated emotions, regarded rather retrospective emotions, such as relief, satisfaction, disappointment, and anger (Baumgartner et al., 2008). Importantly, however, to the best of the authors’ knowledge, anticipatory emotions defined as currently experienced emotions about a future event have not yet been studied within the context of physical activity.

Compared to anticipatory emotions, there is more research on anticipated emotions. In another study, Perugini and Bagozzi (2001) focused on anticipated emotions in exercising and dieting and showed that positive anticipated emotions positively influenced decisions regarding these future behaviors. Comprising social behaviors and decisions, a meta-analysis showed that anticipated emotions can guide social behavior and judgment (DeWall et al., 2015). In exercise psychology, research rather focused on *anticipated affect* than on anticipated emotions. Anticipated affect is defined as “the expectation of how one will feel in response to engaging in, or failing to engage in, physical activity” (Stevens et al., 2020, p. 10). A recently published narrative review indicated a positive association between positive anticipated affect and positive affect during and after exercising and the intention toward exercising (Stevens et al., 2020). However, only one study showed that positive anticipated emotions associated with having successfully engaged in physical activity for 90 days were a stronger predictor for future exercising after this period of time than negative anticipated emotions associated with having failed to engage in physical activity for 90 days (Dunton and Vaughan, 2008). This finding is congruent with previous research on emotions experienced during exercising. As Rhodes and Kates (2015) revealed in a systematic review, positive emotions experienced during exercising are positively associated with future exercise behavior. A more recent systematic review showed that positive emotions, such as enjoyment, can be promoted through intervention studies in order to increase exercise participation (Klos et al., 2020). Included studies referred to positive affect or enjoyment or even used both terms synonymously. However, enjoyment should be understood as a specific emotion while positive affect is used as a more general term in this field of research (Chen et al., 2021). Based on the presented reviews one could conclude that regular exercisers anticipate positive emotions and that the promotion of those lead to a higher level of physical activity. So far, the valence of affect has been a central predictor of future exercise behavior in research (Rhodes et al., 2009; Rhodes and Kates, 2015). Accordingly, it is reasonable to conclude that negative emotions lead to avoidance of the behavior (Higgins, 1997). However, given the complexity of physical activity this perspective might oversimplify the role of emotions in this particular behavior (Baumeister et al., 2007) and the influence of different emotions remains unconsidered.

While some studies focused on the valence of anticipated emotions, studies on specific anticipated emotions, such as regret, pride, and enjoyment present interesting results about how these specific emotions may influence intentions, exercise behavior, and emotions during and after exercising. Two studies focusing on anticipated regret showed that the imagination of failing to exercise predicted intentions regarding exercising and the exercise behavior itself (Abraham and Sheeran, 2003, 2004). Research on self-conscious anticipated emotions, such as pride and shame, in competitive, long-distance runners revealed that training progress was predicted by pride at the within- and between-person level (Gilchrist et al., 2018a), while in another study, neither pride nor shame predicted training effort (Gilchrist et al., 2017). Moreover, expended effort was greater in participants who reported more anticipated pride than others (Gilchrist et al., 2017). In a third study on anticipated pride, more pride was anticipated in participants that were not as fit as they wanted to be compared to their imagined, ideal fitness status when they were asked to anticipate their emotions after a successful sport scenario (Gilchrist et al., 2018b). The anticipation of enjoyment was evaluated in two studies (they used enjoyment and positive affect synonymously) (Ruby et al., 2011; Loehr and Baldwin, 2014). These studies showed that anticipated enjoyment was a significant predictor of experienced enjoyment, and anticipated enjoyment was significantly lower in inactive individuals while experienced enjoyment did not differ between active or inactive individuals (Loehr and Baldwin, 2014). At the same time, both studies also revealed a forecasting error, meaning that anticipated enjoyment was lower than the expected enjoyment during exercising (Ruby et al., 2011; Loehr and Baldwin, 2014). This forecasting error appeared regardless of the type of exercise (Ruby et al., 2011). The presented state of research shows that the number of studies on specific anticipated emotions in exercise behavior is limited, but it gives intriguing insights into how specific emotions can influence exercise behavior.

There are various theoretical positions on how to distinguish between emotions. One model to differentiate emotions was proposed by Ortony et al. (1988; commonly referred to as the OCC-model, Clore and Ortony, 2013). Interestingly, this theoretical approach has also been used by Bagozzi et al. (1998) to assess anticipatory and anticipated emotions (Perugini and Bagozzi, 2001; Baumgartner et al., 2008). According to Ortony et al. (1988), so called emotion types can be differentiated based on the appraisal of conditions. Whether or not an emotion develops depends on the goals (appraisal of desirability), interests (appraisal of praiseworthiness), and beliefs (appraisal of appealingness) of each individual. Additionally, the model acknowledges that emotions are individually constructed and an emotion with the same label (e.g., anxiety) can be experienced differently by individuals (Clore and Ortony, 2013). The authors assume a cognitive structure of emotion types that depend on three major aspects of the world, namely events, agents, and objects. With regards to the first aspect, “events,” emotions can be differentiated depending on whether the outcome of an event concerns the own person (e.g., joy) or someone else (e.g., pity). Furthermore, outcomes can already have happened (e.g., grief) or they can be prospective (e.g., anxiety). In this case, expectations about an outcome can be realized (e.g., satisfaction, fears confirmed) or not (e.g., disappointment, relief). Concerning the second aspect, “agents,” outcomes can be viewed as one’s own praiseworthy (e.g., pride) or blameworthy (e.g., shame) action or as someone else’s praiseworthy (e.g., admiration) or blameworthy (e.g., reproach) action. The third aspect, “objects,” can elicit emotions when objects are appraised as appealing (e.g., love) or unappealing (e.g., hate) (for a more detailed description of the OCC-model see Ortony et al., 1988; Clore and Ortony, 2013).

In summary, both anticipatory emotions and anticipated emotions seem to be two constructs that can contribute to a better understanding of whether an individual engages in exercise or not. Because these two constructs have so far received little attention in exercise psychology, we conducted an exploratory study with two main aims. The first aim was to identify and describe (a) categories of anticipatory emotions experienced before exercise, and (b) categories of anticipated emotions expected to be experienced during and after exercise in regular and non-regular exercisers. Because the experience of emotions has been shown to depend on their measurement either “during” or “after” exercise (Ekkekakis et al., 2011), we referred to both time points for anticipated emotions. The second aim was to develop a theoretical model of anticipated emotions that are particularly related to exercising. For that purpose, the interviews intended to reveal the appraisal processes related with emotions as these appraisal processes seem crucial for the development of the theoretical model. In contrast to the OCC-model as a rather general emotion model, the postulations of the theoretical model target the appraisal processes that are particularly relevant for the context of exercising.

## Materials and Methods

A qualitative study using semi-structured interviews was conducted to explore the role of anticipatory and anticipated emotions regarding future exercise behavior.

### Participants

A purposive sampling strategy was used, considering three different factors to generate a high heterogeneity of the sample: gender (male vs. female), regularity of exercising (regular vs. non-regular), and the type of exercise (individual vs. group). The combination of these three factors built the inclusion criteria to participate in the study. Individuals that could not identify themselves as either regular or non-regular exercisers or participated in individual and group exercises equally were excluded. The participants needed to be between 18 and 65 years old as this study focused on adults. In line with the transtheoretical model (Prochaska and DiClemente, 1983), someone was considered a regular exerciser when their current type of exercise had been carried out at least once a week for at least 6 months. Even though tennis, squash, and climbing can also be regarded as individual sports, the selected participants attended group trainings and were therefore counted as exercises in a group. Participants allocated to “individual” exercising trained on their own, with an online coach, or used a mobile application.

Personal contacts of the interviewer were asked to reach out to people who met the criteria for participating in the study. The interviewer did not know the participants personally prior to the study. Two strategies were applied to recruit participants. A purposive sampling strategy allowed to create a high heterogeneity in the study sample, because differences in future-oriented emotions were assumed to be dependent on the predetermined characteristics. Additionally, theoretical sampling (Bryman, 2016) was applied until no more new categories of future-oriented emotions were reported. Through this procedure, a total of 16 participants (8 female) aged between 20 and 48 years (*M* = 26.63, *SD* = 6.66) were recruited (Table 1). Because we could not find many non-regular exercisers participating in groups, only two non-regular participants exercised in a group. General information about the purpose of the study, study procedure and data protection were sent via e-mail to the participants after ethical approval was obtained by the Ethics Committee of the university. Additionally, participants signed written consent forms prior to the interviews.

**TABLE 1:** Description of study sample.

Gender	Regular active, individual	Regular active, group	Non-regular active, individual	Non-regular active, group
Female	2 (running, horse riding)	2 (volleyball, climbing)	3 (home workouts)	1 (home workouts)
Male	2 (weight training, running)	2 (tennis, running)	3 (home workouts, weight training)	1 (squash)

### Data Collection

Semi-structured interviews were conducted with the participants. Interviews were conducted via Skype due to social distancing orders during the COVID-19 Corona virus pandemic. Participants verbally agreed to having the interview recorded. A semi-structured interview manual was created according to a 32-item checklist (Tong et al., 2007). The interviewer outlined the definition of anticipatory and anticipated emotions before the interview started. The interviews started with general questions followed by more specific questions. The first four questions addressed the participants’ current exercise behavior such as the kind of exercising, how the exercise program was structured and for how long this exercise program has already been carried out by the participant. In three more questions, the participants were asked about their reasons for exercising and how important exercising is for them to estimate the role of exercising the participants’ life. The term exercise is a subcategory of physical activity defined as “physical activity that is planned, structured, repetitive, and purposive in the sense that improvement or maintenance of one or more components of physical fitness is an objective” (Caspersen et al., 1985, p. 128).

Further, the interview was divided into two sections to question the participants about (a) their anticipatory emotions, and (b) about their anticipated emotions during and after exercising. For both sections, the interviewer referred to the type of exercising, which the participants had performed in the last 6 months the most. The interview manual for both sections was structured similarly. After one central question several additional questions followed to gather more information about each specific emotion. Thus, three question catalogs involving a central question and some additional questions were conceived, one question catalog for (a) anticipatory emotions and two for (b) anticipated emotions. The central question in the first section on anticipatory emotions was *“What do you feel right now when you think about your next sport or exercise session?”* In the second section, the central question was about the participants’ anticipated emotions during exercising: *“What feelings are you expecting during the next sport or exercise session?”* In a third central question, the participants were asked about their anticipated emotions after exercising: *“What feelings are you expecting immediately after the next sport or exercise session?”* After each of the three central questions, participants were asked to describe their feelings and related thoughts. They were also asked if a physical sensation was related to this feeling and if they can find a name for this specific emotion. These additional questions were part of all question catalogs. Regarding (a) anticipatory emotions, participants were also asked how this anticipatory emotion is related to similar situations experienced in the past and to the present situation. Regarding (b) anticipated emotions, participants were also asked if they know why they anticipate these specific emotions. This makes a total of seven questions regarding one anticipatory emotion and a total of five questions regarding one anticipated emotion. All questions of the interview manual were asked if they were not already obviously answered beforehand. This was only the case regarding the name of specific emotions (e.g., “I would call this feeling …”). After the interview catalog regarding a specific anticipatory or anticipated emotion was completed, participants were asked if they feel or anticipate any other feelings that are different from the feeling described before. Then the same question catalog was applied again. The full interview manual can be found in the supporting information. Interviews were transcribed verbatim using the f4 transcription software (Dresing and Pehl, 2018).

### Data Analysis

For the organization of the data the f4 analysis software was used (Dresing and Pehl, 2018). Transcripts were analyzed according to the principles of the Grounded Theory (Strauss and Corbin, 1996). This analysis included three steps. Open encoding as the first step in the analysis process started after the first interviews were transcribed. Full texts were coded by building different concepts inductively and classified into categories in order to group similar concepts. In the second step, axial coding was used to find connections between categories. Relationships between categories and subcategories were analyzed within the third step of selective coding. The final categories reflect the anticipatory and anticipated emotions of exercisers. For anticipated emotions, a theoretical model was proposed based on relationships between emotion categories. Additionally, anticipated emotions were embedded in a broader context resulting in a process model. The statements were interpreted with regards to existing emotion theories. For example, when participants reported only the valence (feeling good or bad) or the arousal (feeling relaxed or tensed) of an emotion, these statements were not interpreted as distinct emotion categories. Direct quotations in the section “Results” serve as examples and were analogously translated into English by the first author of the study.

### Trustworthiness

We applied several steps to increase the trustworthiness of the present study. The interviewer was a graduate in sports and health sciences with personal experiences in competitive sports. Her experience in various team and individual sports helped her understand the different perspective of the participants. Social desirability bias (Bergen and Labonte, 2020) can occur in interviews about sensitive topics such as emotions. Therefore, interviews were conducted with only one researcher and participants were informed about the study topic and aim prior to the interview. Furthermore, the interviewer did not know the participants personally before the interviews, allowing for a neutral relationship between the interviewer and the participant. With this procedure we aimed to reduce the risk of social desirability bias and increase the trustworthiness of the participants’ statements.

Interviews were transcribed by the first author, and the transcripts were then checked by the participants. During the inductive coding process, some of the participants’ statements remained unclear for interpretation. Therefore, member reflection conversations were conducted with six participants in total. Member reflection conversations were undertaken to gain additional data and to clarify ambiguous descriptions of emotions in the interviews (Smith and McGannon, 2017). Semi-structured interview manuals were individually created for each member reflection conversation based on the first interviews. When participants had difficulties describing an emotion or were unsure how to differentiate emotions from each other, the interviewer supported the participant with examples of emotion types in accordance with the OCC-model (Ortony et al., 1988). In the interpretation process of the participants’ statements, other researchers in the area of sport and exercise psychology served as critical friends. The results drafted by the first author were critically discussed to reflect on alternative interpretations of the interview statements and to improve the comprehensibility of the results.

## Results

### Anticipatory Emotions

Seven different emotion categories emerged through the interpretation of the participants’ reported anticipatory emotions. All participants stated at least one and up to three different anticipatory emotions.

#### Enjoyment

Ten participants, of which five exercised in a group or with a partner, and seven were males, reported joy, enjoyment, and happiness when thinking about the next exercise session. Statements about having fun were also summarized under the term enjoyment. Seven out of ten participants were regular exercisers who looked forward to exercising because the execution of the sport itself was perceived as enjoyable. Participants described this emotion with excitement in the stomach, beaming with joy and smiling when thinking about their sport. Enjoyment was attributed to meeting teammates and friends with whom exercising is more fun. Participants stated that a short period of abandonment due to injuries or closures of sport facilities resulted in enjoyment, because they were looking forward to executing their sport again. Other reasons were that exercising was perceived as a change to the daily working routine, as an experience of nature, or as a possibility to increase the performance level.

*“In that regard, I’m happy that I can be active again and do something good for myself”* (No. 7, male, non-regular exerciser, individual).

*“Yeah, it plays an extremely big role for me when I’ve had a stressful week at work, and that’s very common. The training is on Friday, it’s this compensation really, this coming down and this shaking the stress off. That’s actually the greatest joy”* (No. 4, female, regular, group).

#### Anxiety

Five exercisers, of which two were regular exercisers and three were non-regular exercisers, recognized being anxious when thinking about their next exercise session. All of them exercised on their own and four out of five participants were female. They reported being afraid of disappointing themselves with a bad performance and harming their body physically. Furthermore, they experienced themselves as not being good enough compared to their former fitness level or the fitness level of others. Some statements also indicate that anxiety may be an emotion that precedes the development of disappointment or shame. Additionally, participants showed physical symptoms of anxiety such as a queasy feeling in the stomach and an uncontainable feeling of nervousness.

*“Also, a little bit of anxiety, because right now or a few weeks ago I had a little bit of trouble with running”* (No. 3, male, regular exerciser, individual).

*“(*…*) that’s why it’s also a bit of fear of seeing that what I achieved was all for nothing. And that’s just demotivating”* (No. 5, female, non-regular exerciser, individual).

*“Yes, it goes in the direction of fear. I can’t describe the queasy feeling in my stomach very well, it just comes, and I can’t control it. But it’s just so uncomfortable what’s going on in my stomach. Then, I also get nervous and just don’t want to go outside or move at all”* (No. 12, female, non-regular exerciser, individual).

#### Relief

One female regular exerciser reported being relieved when thinking about her next exercise session. Her perceived relief was due to a full recovery after a long period of injuries and being able to train again. This participant was part of a volleyball team as a player and a coach.

*“The fact that it’s working again now, that I have fought my way back (to playing volleyball), is just such a relieving feeling, which I still have when I think about it”* (No. 2, female, regular exerciser, group).

#### Pride

One male non-regular exerciser was glad that he had been physically active in the past, so that he had a fitness level to build on. He was not grateful for something that was given to him but rather proud that he exercised in the past which is a benefit for him now.

*“To some extent maybe, when I look back on past exercise trainings, then some of it is gratitude toward myself for exercising back then”* (No. 16, male, non-regular exerciser, individual).

#### Remorse

One male non-regular exerciser had feelings of remorse, such as feeling guilty about not having exercised regularly in the past. Exercising regularly may be something that others expect from him because as a healthy student without a stressful job he had no excuse not to exercise. This participant used to go on his own to the gym for weight training.

*“Yes, I feel a bit bad about it (not exercising). I have a guilty conscience*. (…) *Yes, I should go to the gym more often, and that it’s good for me, when I go to the gym. I don’t know why I don’t go more often; even though I have time”* (No. 7, male, non-regular exerciser, individual).

#### Shame

One female non-regular exerciser reported feeling shame when thinking about the next exercise session. She reported societal pressure as the reason why she thinks her performance is embarrassing. According to her description, the image of a young woman nowadays involves being athletic. Because she cannot fulfill these expectations, she prefers to exercise alone using online videos.

*“Maybe a little bit of shame. Exactly, because I think socially it’s regarded that it’s a good thing to be athletic”* (No. 6, female, non-regular exerciser, group).

#### Regret

Three regular exercisers stated that they would miss the exercise experience when they imagine not being able to participate in the next exercise session. One female exerciser had strong feelings of regret, because she may leave the team when she is pregnant.

*“If I didn’t do it, I would absolutely miss it”* (No. 2, female, regular exerciser, group). *“Because I know what it’s like when training is canceled or when you don’t have that equalizer on Fridays. That really drags on into half the weekend until you really come down”* (No. 4, male, regular exerciser, group).

*“(*…*) it’s because I would miss something when I wouldn’t do it”* (No. 14, male, regular exerciser, group).

### Anticipated Emotions During Exercising

Emotions that were anticipated to be felt during the next exercise sessions were transformed into ten different emotion categories. All participants reported at least one and up to four different anticipated emotions during their next exercise session.

#### Enjoyment

Enjoyment was described by eleven participants, of which six were regular and five non-regular exercisers. Four out of eleven participants exercised in groups, and six exercisers were male. Enjoyment was the most frequently reported anticipated emotion during the exercise session. Five participants explained that they anticipated to enjoy the next exercise session because they like executing the sport and experience it as fun. Enjoyment was also described as an exhilaration or as an exuberant feeling of joy. This emotion was accompanied by laughing, joking, feelings of ease and strength during the training.

*“Enjoyment, enjoying the sport. I go into the climbing hall and I’m just insanely happy to be able to do the sport”* (No. 9, female, regular exerciser, group).

*“I laugh and have fun, because I’m joking with others”* (No. 2, female, regular exerciser, group).

*“So, it’s a very, very positive feeling I must say, at this moment. I’m really looking forward to it*. (…) *At first, it’s pure joy”* (No. 1, female, regular exerciser, individual).

*“At the beginning, in any case, very happy and glad about doing it* (…)*”* (No. 8, male, non-regular exerciser, group).

#### Displeasure

One female and two male non-regular active participants anticipated to feel displeasure during their next exercise session which is why they would try to avoid the participation. They were demotivated to engage in future exercise sessions.

*“It’s this displeasure, the avoidance of uncomfortable situations”* (No. 6, female, non-regular exerciser, group).

*“When I realize that I have gotten weaker and I don’t feel strong during the exercises, I become demotivated”* (No. 10, male, non-regular exerciser, individual).

#### Hope

One male non-regular exerciser who trained on his own stated he anticipated to feel hope during his next exercise session. He anticipated to hopefully reach the end of the exercise session and to accomplish a valuable task.

*“(*…*) or maybe also hope in the sense that soon I will have made it and will have done something good”* (No. 16, male, non-regular exerciser, individual).

#### Anxiety

The emotion anxiety was reported by seven participants, two non-regular, and four regular exercisers. Four participants were female and three participants exercised on their own. Anxiety or nervousness was anticipated to be felt during their next exercise session, because they expected some undifferentiated negative consequences, such as failing certain tasks. One participant stated that the change of environmental conditions could compromise her safety.

*“(*…*) sometimes you have thoughts of fear or what would you do when you can’t control your horse*. (…) *You don’t know what could happen or you are afraid that something could happen that you can’t anticipate”* (No. 11, female, regular exerciser, individual).

*“So, at the beginning, I would say that the first 5–10 min are pretty exciting”* (No. 4, male, regular exerciser, group).

#### Satisfaction

Three non-regular and four regular active participants anticipated to feel satisfaction during their next exercise session. Five out of seven exercisers who anticipated satisfaction participated in groups and four of the seven participants were male. The satisfaction was related to the participants’ own ability to participate in the training or the positive outcome of a match. Participants used the terms calmness and detachedness to describe their feelings. Some participants closely linked the experience of satisfaction to the emotions enjoyment and pride.

*“I’m satisfied that I decided to do it again* (…)” (No. 13, female, non-regular exerciser, individual).

*“A certain satisfaction and great joy when you have won and rewarded yourself for the work during the session”* (No. 8, male, non-regular exerciser, group).

*“I would say this interaction gives you, somehow, satisfaction”* (No. 4, male, regular exerciser, group).

#### Disappointment

Two non-regular active participants reported to expect disappointment during the next exercise session. The emotion was directed to themselves because they anticipated consequences, which they tried to avoid. Both participants also said that the emotion self-anger accompanied the emotion disappointment.

*“Yes, a certain sense of disappointment is already there if it doesn’t get better or doesn’t work out, even though you tried with all your strength to change something* (…)” (No. 8, male, non-regular exerciser, group).

*“(*…*) when I don’t achieve the things that I’ve set out to reach or when I don’t have the power that I would have had for that exercise, then I’m really disappointed with myself and maybe a bit angry, too”* (No. 12, female, non-regular exerciser, individual).

#### Anxiety Confirmation or Despair

One non-regular exercising female participant anticipated that her fears would come true which would result in despair. She expected to fail in some exercise tasks and she did not know how to cope with this situation. Despair was related to frustration and self-anger.

*“(*…*) the fear that I’m no longer able to accomplish certain tasks and of being desperate*. (…) *I will probably be desperate in that moment, because I’m not able to execute the task”* (No. 13, female, non-regular exerciser, individual).

#### Pride

Three female and two male non-regular exercisers who trained individually anticipated to feel pride during the next exercise session. On the one hand, they were proud of themselves because of their decision to participate in the exercise session and on the other hand, they linked pride to the experience of success. In the view of some participants, the experience of pride was closely linked to enjoyment.

*“A little bit proud, because I managed to overcome my weaker self, again*, (…) *as I said, pride, because I did it again* (…)” (No. 5, female, non-regular exerciser, individual).

*“Actually, joy and pride that I made it”* (No. 7, male, non-regular exerciser, individual).

*“Depending on how much you’ve done, you’re prouder than you would be if you’d only done one fitness video”* (No. 6, female, non-regular exerciser, group).

*“I think it feels a bit like success when I exercise, because I notice that it really feels good and it feels like happiness* (…)” (No. 13, female, non-regular exerciser, individual).

#### Self-Anger

Five participants, of which two were regular and three were non-regular exercisers, anticipated to feel self-anger during their next exercise session. Two of them exercised in groups, and three out of five participants were females. Self-anger was anticipated due to experiences of failure and the self-evaluation of being unable to fulfill the tasks. Two participants linked their self-anger to the feeling of frustration.

*“If I stop, I have to be really angry or frustrated, then, I stop and that’s the point where I say okay, I’m not running any further now”* (No. 1, female, regular exerciser, individual).

*“I also have phases where I’m concentrated, where I get angry, because something doesn’t work out the way I would like it to* (…)” (No. 2, female, regular exerciser, group).

*“Yes, depending on how stupid the loss of a point or the final result was or how unfortunate it was, I would like to smash the racket on the ground from time to time. But of course, you can’t do that”* (No. 8, male, non-regular exerciser, group).

*“It has happened that I’ve been angry at myself and that lead to the fact that I just give up and I don’t continue the exercise session”* (No. 12, female, non-regular exerciser, individual).

#### Shame

One female non-regular exerciser described the fear of getting exposed in front of others while exercising. In a member reflection conversation, she described this feeling with the emotion shame. To reduce this negative emotion, she predominantly exercises at home and avoids the attention of others.

*“I just feel extremely uncomfortable in a gym, and these feelings of worry and fear would be even bigger than they already are at home”* (No. 12, member reflection, female, non-regular exerciser, individual).

### Anticipated Emotions After Exercising

Based on the anticipated emotions after exercising reported by the participants, eight different emotion categories were conceptualized. All participants stated to feel at least one and up to five different anticipated emotions after the next exercise session.

#### Enjoyment

Eight participants, of which four were regular and four were non-regular exercisers, anticipated to feel enjoyment after their next exercise session. Two of them participated in a group and six were males. The exercisers who anticipated enjoyment expected to enjoy the physical and mental state after exercising. Participants described the anticipation of enjoyment as a consequence of the fun they would be experiencing during the exercise sessions. They anticipated that enjoyment would influence their general mood and attitude positively. This effect was supported by evaluating the exercise session as successful.

*“I expect, and it occurs like that almost every time, that I feel great. I’m happy and directly after I stopped running in the forest, I experience joy after work is done”* (No. 1, female, regular exerciser, individual).

*“It is in any case then simply the enjoyment to have played again, to have exercised again, to have moved again and simply to have had fun”* (No. 8, male, non-regular exerciser, group).

*“Predominantly I’m glad; joyous that I did it”* (No. 16, male, non-regular exerciser, individual).

*“Being happy and having joy in having achieved that”* (No. 12, female, non-regular exerciser, individual).

#### Displeasure

One non-regular male exerciser expected to quit early from exercising because he anticipated to experience displeasure during the exercise session.

*“When it (the exercise session) didn’t go that well, it wasn’t fun and exhausting, then I quit. I say to myself, it just doesn’t make sense today, and I don’t feel pleasure”* (No. 10, male, non-regular exerciser, individual).

#### Hope

One male non-regular active participant anticipated to feel hope besides enjoyment, after his next exercise session. Based on the assumption that the next exercise session may be a positive experience, he anticipated having hope for becoming a regular exerciser.

*“Maybe also a little bit of hope that this overcoming which I had to put up with before, was perhaps not so great and this should get me to exercise more regularly in the future*. (…) *besides the joy that I did it, I think that I should do it more often and that it mainly was a positive experience”* (No. 16, male, non-regular exerciser, individual).

#### Satisfaction

Nine participants, of which four were non-regular exercisers stated that they anticipated to feel satisfaction after their next exercise session. Five out of nine exercisers participated in a group, and four exercisers were females. Satisfaction was the most frequently anticipated emotion after exercising. Participants anticipated to be satisfied with their physical effort during the exercise session and with benefits associated with the exercise session (e.g., meeting friends). Three participants closely linked the emotion satisfaction to the emotions enjoyment and pride.

*“Well, satisfaction, just to have burned off energy, to have given everything, to have met friends, just to have had a great evening and then you go home satisfied”* (No. 9, female, regular exerciser, group).

*“Yeah so, satisfaction and joy. I’m actually happy afterward. Happy and satisfied*. (…) *Exactly, so as I’ve said, the satisfaction with myself is quite big”* (No. 7, male, non-regular exerciser, individual).

*“(*…*) I would feel satisfied again, maybe a little exhausted depending on how I exerted myself, but in general I would feel satisfied, I assume”* (No. 13, female, non-regular exerciser, individual).

*“You are simply satisfied that you’ve done something. That you have moved, and that you already know, from the past, that what you have just done is good for you”* (No. 14, male, regular exerciser, group).

In a member reflection conversation, one participant explained that anticipated satisfaction depends on the success of his own performance. He then referred to the emotion pride. Two other participants specified their emotions as anticipated satisfaction in member reflection conversations.

#### Disappointment

One male regular exerciser and one male non-regular exerciser anticipated to be disappointed after their next exercise session. They anticipated this emotion for when the performance outcome is evaluated as poor or not good enough resulting in a feeling of dissatisfaction.

*“Probably also sometimes disappointment, if it didn’t go so well”* (No. 3, male, regular exerciser, individual). *“Yes, dissatisfaction*. (…) *I have not shown the performance I would have expected”* (No. 10, male, non-regular exerciser, individual).

#### Relief

Four participants, of which two were non-regular and two were regular active exercisers anticipated relief after their next exercise session. Three out of the four participants exercised on their own and three participants were males. The exercisers anticipated to be relieved because eventual negative consequences of their performance may not occur. Doubts which they had prior to the exercise session have would not be confirmed. A non-regular active participant anticipated to be relieved, because he would not have to exercise again, as he already did a training session this week.

*“Just like you can check something off from a to-do list. Relieved, just like relief. The relief of doing a task that you don’t like so much, but have the feeling that you have to do it”* (No. 6, female, non-regular exerciser, group).

*“Well, I mean, to some extent you’re always glad when training is over. I like it very much, but I’m also happy when it’s over because it’s a really big effort for the body. I’m happy when I’m coming home relieved”* (No. 15, male, regular exerciser, individual).

*“Relief comes up when I can estimate or foresee the end of the training and I’m convinced that this initial nervousness or fear of failure will not occur”* (No. 14, member reflection, male, regular exerciser, group).

#### Pride

Five participants, of which four were non-regular active exercisers, anticipated to feel the emotion pride after the next exercise session. Three out of five exercisers were females, and four participants exercised individually. The exercisers explained that they anticipated to be proud of themselves for taking the step to participate in the exercise session or for accomplishing more during the training than they had expected. In one of the exerciser’s descriptions, the anticipation of pride was related to the achievement of personal goals.

*“So, the positive feelings after exercising are that I’m happy that I did it again. I’m actually proud of myself and feel real joy, and think to myself, wow, good!”* (No. 5, female, non-regular exerciser, individual).

*“Depending on how much you’ve achieved, you’re prouder than you would be, if you had only done one exercise video”* (No. 6, female, non-regular exerciser, group).

*“I’m very proud of myself that I managed to go to the gym and pull it off”* (No. 7, male, non-regular exerciser, individual).

*“It’s less pride toward others, so it’s not like that you’ve accomplished some work, but pride in the sense that even though it is very limited running, you’re setting personal goals”* (No. 14, male, regular exerciser, group).

Another female non-regular exerciser described feelings of hubris as a sense of superiority over others and a heightened form of pride. She anticipated becoming more athletic over the next exercise sessions while others remain inactive even though she was a non-regular exerciser herself.

*“Maybe you can call it a bit of arrogance, that I think yes, I’ve made it* (…), *I feel really good now, because I’ve achieved more than someone else”* (No. 12, female, non-regular exerciser, individual).

#### Self-Anger

Two participants reported that they anticipated becoming angry with themselves after their next exercise session. One female non-regular exerciser anticipated the emotion of self-anger, because she expected herself to perform poorly during the exercise session and explained this with non-regular training, although she is aware of the benefits of physical activity. Another female but regular exerciser would only anticipate this emotion if she were to have performed poorly that day. This indicates that self-anger depends on the performance outcome of the exercise session. The emotion was reported to be expressed through nervous movements and harsh self-talk.

*“(*…*) I know that it is important that you are physically active, and then I get angry at myself that I’m so exhausted from jumping around for 20 min*. (…) *Yes, anger and rage at myself for being such a loser* (…)” (No. 5, female, non-regular exerciser, individual).

*“Well, should it not go well, maybe also anger. So rather a negative emotion, when I realize oh, well, the bouldering session today did not work out at all* (…)” (No. 9, female, regular exerciser, group).

### Theoretical Model of Anticipated Emotion Categories

**FIGURE 1:**
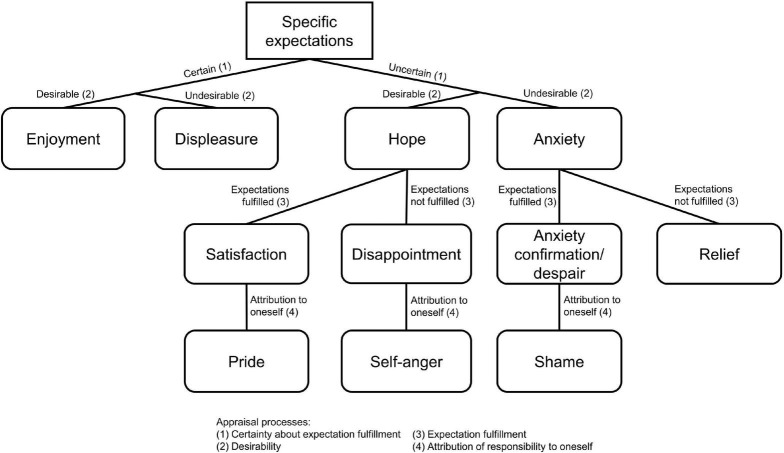
Theoretical model of anticipated emotion categories in exercise behavior.

Based on the identified emotion categories, a theoretical model of anticipated emotion categories was derived, including anticipated emotions during and after exercising (Figure 1). This theoretical model postulates that different emotion categories are anticipated depending on specific appraisal processes.

Four appraisal processes that guide the anticipation of emotions have been identified from the interviews: (1) certainty about expectation fulfillment, (2) desirability, (3) expectation fulfillment, and (4) attribution of responsibility to oneself. The occurrence of emotion-provoking events during exercise can be appraised as either relatively certain or relatively uncertain. If an individual is convinced that the expectation will come true, they anticipate enjoyment when the outcome is desirable or displeasure when the outcome is undesirable. For example, one exerciser anticipated enjoyment because she was convinced that it would be fun to meet friends and to play as a team (No. 2). Another participant anticipated feelings of displeasure while exercising because she had multiple negative experiences and so, expected negative consequences for sure (No. 6). In the case of uncertain outcomes, one anticipates hope if the consequences are expected to be rather desirable or anxiety if the consequences are expected to be rather undesirable. One participant anticipated to feel hope during exercising because he was unsure if he would persevere until the end of the session (No. 16), while another participant anticipated to feel anxiety because she was unsure about possible negative consequences that would come up during the exercise session, such as being judged by others (No. 6).

Hope and anxiety are emotions with a prospective character and depend on the valence of the expected consequences. Depending on the actual consequences, emotions with a responsive character can develop. The development of emotions with a responsive character is driven by the anticipation of the fulfillment of expectations. In the case of hope, when the individual expects desirable consequences during or after the exercise session, the anticipation that these consequences actually come true can be associated with the anticipation of satisfaction. In contrast, if the individual anticipates that the desirable consequences will not occur, they would rather anticipate disappointment. For example, one participant anticipated to be disappointed because she hoped for positive performance outcomes even though she did not exercise regularly and it was likely that her desirable consequences would not occur (No. 12). Likewise, in the case of anxiety, when the individual expects undesirable consequences during or after the exercise session, the anticipation that these consequences actually come true can be associated with the anticipation of despair. For example, one exerciser anticipated anxiety, resulting in despair because the upcoming exercise tasks would be too hard (No. 13). In contrast, if the individual anticipates that the undesirable outcomes will not occur, they would rather anticipate relief. One participant explained that relief occurs after the training because the expected fear of failure during the running session did not arise (No. 14).

Finally, the outcomes are appraised in terms of their causes. In the interviews, individuals frequently attributed the causes of outcomes to themselves and this attribution process was related to the formation of three emotions: pride, self-anger, and shame. First, if one attributes anticipated desirable consequences to the competence of oneself, anticipated satisfaction can develop into anticipated pride. For example, one participant anticipated to be proud after training because he decided to participate until the end and take care of his physical health (No. 7). Second, if an individual blames oneself for the unexpected undesirable consequences, anticipated self-anger can emerge from anticipated disappointment. One participant anticipated to feel anger and rage after exercising because she attributed the physical exertion to her insufficient performance level (No. 5). Third, if an individual blames oneself for the expected undesirable consequences, anticipated shame can arise from anticipated despair. For example, one participant anticipated to feel shame because she feels embarrassed when exercising in front of others (No. 12). It should be mentioned that the emotion categories anxiety confirmation/despair and shame were only anticipated by one participant, respectively.

## Discussion

The main aim of this study was to identify categories of anticipatory emotions and categories of anticipated emotions during and after exercising. Categories of anticipatory emotions were enjoyment, anxiety, relief, pride, remorse, shame, and regret. Categories of anticipated emotions during and after exercising were enjoyment, displeasure, hope, anxiety, satisfaction, disappointment, anxiety confirmation/despair, relief, pride, self-anger, and shame. The valence of anticipatory and anticipated emotion categories was positive as well as negative within both regular and non-regular exercisers. However, tendencies show that regular exercisers anticipated more positive emotions, while non-regular exercisers anticipated more negative emotions. This tendency was more apparent for anticipatory emotions. The results revealed not only a detailed description of emotion categories, but also allowed to conceive a theoretical model of anticipated emotion categories which may help to explain how different emotion categories emerge based on expectancies and appraisal processes.

### Anticipatory Emotions

The results of this study demonstrate that anticipatory emotions can be clustered into three groups. First, anticipatory emotions, such as pride, relief, shame, and remorse were based on past experiences with exercising, but were still viewed as relevant for future exercising. Participants felt proud or guilty because they had or had not been exercising for a while, they felt ashamed the last time they exercised, and they felt relieved after long period of injuries. Second, the emotion regret is different from the other emotions because it involves the imagination of missing the next exercise session. All participants that reported regret were regular exercisers, which fits with the results published by Abraham and Sheeran (2003, 2004) who showed a positive association between regret to miss an exercise session and exercise behavior.

Third, the anticipatory emotions enjoyment and anxiety were directly related to events during the next exercise session. Compared to pride, relief, shame, and remorse, these two emotions were not only based on past experiences but also on expected events such as meeting friends or experiencing social pressure specifically during the next exercise session. According to the OCC-model (Ortony et al., 1988), enjoyment can be experienced when one is pleased about a desirable event. Participants stated that they execute their sport primarily because they enjoy it. Therefore, enjoyment was viewed as an emotion that “leads to performing an activity primarily for its own sake” (Kimiecik and Harris, 1996, p. 258) which is closely linked to intrinsic motivation (Ryan and Deci, 2000; Wienke and Jekauc, 2016). According to the Self-Determination Theory, enjoyment is postulated as a relevant regulatory process that determines intrinsic motivation “which refers to doing an activity for the inherent satisfaction of the activity itself” (Ryan and Deci, 2000, p. 71). We identified the need of autonomy, competence, and relatedness as important factors whose satisfaction could have enhanced enjoyment and subsequently the intrinsic motivation of the participants in the present study. Several participants reported that meeting friends, increasing performance, and reducing stress are important aspects for experiencing enjoyment. Anxiety has a prospective character because the undesirable consequences are anticipated and have not yet happened (Ortony et al., 1988; Baumgartner et al., 2008). As outlined in previous studies, depending on the outcome of the event, anxiety can be an antecedent of emotions with a responsive character (Ortony et al., 1988; Baumgartner et al., 2008). With regards to the definition of anticipatory emotions (Baumgartner et al., 2008), these emotions were experienced in the moment of the interview but refer to a future event. Thus, it is important to consider that a current emotional experience can also be influenced by other factors, like one’s current mood.

### Anticipated Emotions

During the analysis process of anticipated emotion categories, it was notable that both regular and non-regular exercisers anticipated positive as well as negative emotions. However, it must be mentioned that regular exercisers described more positive anticipated emotions and non-regular exercisers described more negative anticipated emotions. Additionally, participants reported a greater variety of anticipated emotions compared to anticipatory emotions. In a previous study examining anticipatory and anticipated emotions related to the historic event of the millennium change, it was postulated that anticipatory emotions typically have a prospective character (hope, anxiety), while anticipated emotions typically have a responsive character (e.g., disappointment, satisfaction) (Baumgartner et al., 2008). However, in our study, we found that anticipated emotions can have both a prospective and responsive character. The event of an exercise session must be distinguished from historic events, such as the millennium. The results of the present study underline the uniqueness of an exercise session that extends over a limited period of time and that contains several small events which can elicit different emotions compared to the imagined consequences of one historic moment. Moreover, anticipated emotions involve a greater cognitive component (Williams et al., 2019). While anticipatory emotions follow automatic pathways, anticipated emotions are part of a more reflective process (Stevens et al., 2020).

The guiding role of anticipated pleasure or displeasure in decision making has already been investigated years ago. Mellers and McGraw (2001) suggest that individuals choose their behavior with the highest likelihood of it being pleasurable compared to other options. Furthermore, they acknowledge that anticipated pleasure can increase when individuals anticipate their performance to be high and satisfying. In the present study, especially non-regular exercisers were unsure of what to expect from the next exercise session because they could not estimate their own abilities and tended to overstrain themselves. The level of effort of the exercises should match the current abilities and needs of the participants in order to promote outcome expectancies appraised as desirable. As shown in various studies, exercising with moderate intensity is perceived as more pleasurable than high intensity exercise (Ekkekakis, 2003; Ekkekakis et al., 2004, 2011), suggesting that matching skills to training demands promote positive affect and desirable outcome expectations. In particular, female, non-regular exercisers expected negative outcomes because their physical abilities were estimated as low compared to the demands of the workout, and they anticipated rather negative emotions, such as displeasure, anxiety, disappointment, shame, and self-anger. Literature suggests that higher levels of social media use can predict increased body shame (Salomon and Brown, 2018) and reduce motivation to exercise (Robinson et al., 2017). These findings underline the importance of exercise environments in which participants feel secure, respected and encouraged, regardless of their exercise level. It should be pointed out here that the sample of this study consists mainly of young adults (*M* = 26.63, *SD* = 6.66) and that self-conscious emotions were less reported by participants older than 35 years. In addition, a coach could help to motivate exercisers complete their workout, correct wrong movements and set an appropriate exercise load (Strauch et al., 2019).

Parallels to the OCC-model (Ortony et al., 1988) can be found regarding the appraisal processes and emotion categories. As we outlined in the theoretical model of anticipated emotion categories, the development of emotions underlies some sorts of appraisal processes, which are part of many emotion theories (Ortony et al., 1988; Moors, 2009; Scherer, 2010). In the present study, these were (1) certainty about expectation fulfillment, (2) desirability, (3) expectation fulfillment, and (4) attribution of responsibility to oneself. The past three appraisals were part of the OCC-model, resulting in similar emotion categories. In terms of the desirability and expectation fulfillment, Ortony et al. (1988) outlined that satisfaction emerges when the desired consequences occur, while disappointment emerges when they do not occur. In contrast, when expected undesirable consequences come true, anxiety is confirmed whereas relief emerges when they do not come true. Although we did not separate event-based from agent-based emotions, as postulated in the OCC-model (Ortony et al., 1988), the attribution of responsibility was an important condition for the anticipation of pride, self-anger, and shame. Participants reported anticipated pride along with anticipated satisfaction, with the difference that for pride, the outcome was attributed to their personal competence. The same principle applies for self-anger and shame, where participants blamed themselves for failing to prevent undesired consequences. According to the OCC-model (Ortony et al., 1988), the development of self-conscious emotions, such as pride and shame depends on the fulfillment of certain standards in form of values. One’s standards might involve a certain fitness status, and if the value is not achieved, deficits are attributed to oneself. We can find a similar approach in Carver and Scheier’s Control-Process View (Carver and Scheier, 1990) in which they argue that the comparison between the reference value and the current state results in affective reactions and behavior consequences, aiming to reduce the discrepancies. Pride, for example, was anticipated especially by female non-regular exercisers. They were proud of their decision to exercise and to not give up. In addition, they anticipated more pride when more exercises or workout clips were done. A previous study found that anticipated pride may be associated with an increasing training effort (Gilchrist et al., 2017), but this may not apply to inactive individuals. In the present study, participants indicated that anticipated pride was related to social acceptance. Participants reported assessing their current fitness level as far away from the ideal fitness level given by the society or peer-group. When they succeed in overcoming the discrepancies between the current and the social standards, this then would result in the experience of pride (Tracy and Robins, 2004). The anticipation of pride reflects a more external motivation to gain attention and praise. Considering the maladaptive effects of such a rather extrinsic motivation (Ryan and Deci, 2000), the promotion of anticipated pride as an incentive for behavior change is only advisable when goals are based on more autonomous behavior regulations.

**FIGURE 2:**
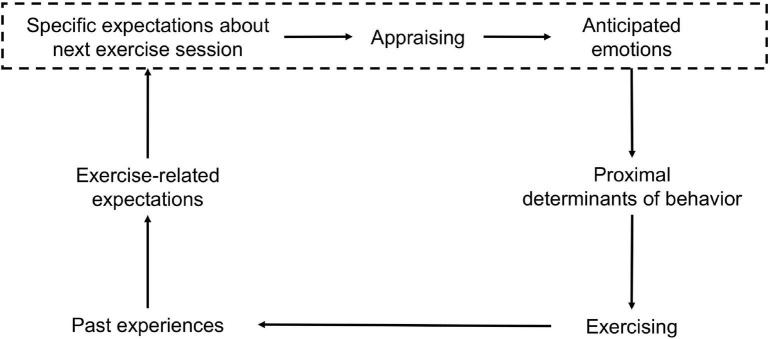
Role of anticipated emotions in exercise behavior.

The outlined theoretical model of anticipated emotion categories focuses on the development and definition of emotion categories based on the relevant appraisal processes revealed in the interviews. Our data give us reason to assume that the development of anticipated emotions (blue box) could be part of cyclical processes in the context of exercise behavior (Figure 2).

As we pointed out in the introduction, expectations can be understood as beliefs about future states based on past experiences (Roese and Sherman, 2013). In the context of the present study, expectations would be exercise-related. We assume that individuals construct specific expectations about their next exercise session based on more general exercise-related expectations. In the present study, specific expectations about the next exercise session mainly referred to one’s own performance level, meeting friends, experiencing social pressure, or being physically challenged. These specific expectations were evaluated by appraisal processes (see Figure 1: certainty about expectation fulfillment, desirability, expectation fulfillment, attribution of responsibility to oneself), resulting in concrete anticipated emotions. Previous research gives reasons to believe that anticipated emotions may not directly influence the decision for or against exercise behavior (Perugini and Bagozzi, 2001; Loehr and Baldwin, 2014; Helfer et al., 2015). Proximal determinants could mediate the influence of anticipated emotions on exercising. For example, studies about anticipated affect in the context of physical activity suggest that anticipated affect influences exercise behavior through intention (Perugini and Bagozzi, 2001; Loehr and Baldwin, 2014; Helfer et al., 2015). However, dual-process approaches show that besides a deliberative and reflective system (e.g., intention, self-efficacy) an automatic and non-reflective system (e.g., habits, affective responses) can influence exercise behavior as well (Brand and Ekkekakis, 2018; Strobach et al., 2020). Finally, as shown in Figure 2, we assume that the actual behavior (i.e., exercising or not) may then influence our experience and future exercise-related expectations.

### Strengths and Limitations

The present study has several strengths and limitations. The study focused on affective constructs that are increasingly gaining attention in exercise psychology and may contribute to a better understanding of why individuals are active or not (Stevens et al., 2020). Additionally, a theoretical model of anticipated emotion categories in exercise behavior derived from the interviews which helps to understand how anticipated emotions develop based on appraisal processes. The purposeful sampling strategy made it possible to find meaningful differences in the responses regarding the regularity of exercising. Member reflection conversations were conducted to reduce misinterpretations. Additionally, critical friends contributed to the quality of the interpretation. However, it is possible that not all relevant future-oriented emotions were identified in the present study. One reason could be that participants did not report all emotions they experienced or anticipated because they could not find the words to do so. Moreover, anticipatory emotions could have been influenced by other factors so that these emotions may have not only regarded the next exercise session but rather exercising in general at the moment of the interview. Another reason could be that participants described their emotions about exercising more positively than they actually were due to a social desirability bias (Bergen and Labonte, 2020). The sample size turned out rather small because a theoretical sampling strategy was applied. This means that participants were recruited according to the set inclusion criteria until no more new categories of future-oriented emotions were reported. It is also important to mention that that sample consisted mainly of young adults restricting the results to this age group. Therefore, caution is advised when interpreting the results beyond the described sample.

### Future Research

The results of the present study and the current state of research point to several intriguing future research directions. First, previous studies in exercise psychology focused on the valence of anticipated affect (Dunton and Vaughan, 2008; Wang, 2011), but our results suggest that distinct emotions may explain important differences in exercise behavior. As an example, further studies on self-conscious emotions, such as pride and shame related to future exercise behavior, especially in non-regular exercisers, would add to the current state of research. In particular, a sample of adolescents and young adults would be appropriate in the context of self-conscious emotions as the relevance of different emotions could change in older age through experiences and a shift of values. Second, the derived theoretical model of anticipated emotions can help to understand relevant appraisal processes related to specific emotions. The appraisal processes outlined in this theoretical model may help understand the emotional processes that are responsible for an individual to exercise or not. Moreover, the theoretical model could be useful for coaches and sport psychologists working with athletes on emotional processes. Third, based on the appraisal processes identified in this study, questionnaires could be developed that may test the postulation of the theoretical model in a larger sample. In the same vein, longitudinal studies seem promising to understand the actual impact of anticipated emotions on exercise behavior. So far, only one study evaluated the association between anticipated affect and the likelihood of physical activity adoption and maintenance (Dunton and Vaughan, 2008), and only a few studies focused on the mediating role of intention between anticipated affect and physical activity (Perugini and Bagozzi, 2001; Loehr and Baldwin, 2014; Helfer et al., 2015). Finally, ecological momentary assessment (EMA) methods offer the opportunity to measure exercise behavior in real-time and allow researchers to assess multiple variables on different time occasions in order to identify antecedents and consequences of exercising (Dunton, 2017). Changes over time in anticipated emotions could be assessed with EMA methods to analyze if decisions for or against participation rely on anticipated emotions. This method could be particularly useful for assessing anticipated emotions occurring one or more days prior to actual physical activity behavior and relating them to actual physical activity behavior. Another interesting methodical aspect would be to assess participants’ facial expressions to take them into account when analyzing participants’ statements about emotions. Previous research suggests that negative affective exercise valuations were related to negative facial expressions on exercise-related stimuli especially in less active people (Brand and Ulrich, 2019). Thus, the application of facial expression recognition (e.g., in form of a software) could increase the rigor and quality in qualitative emotion research.

## Conclusion

This qualitative study provided insights into anticipatory and anticipated emotions as two future-oriented affective constructs related to exercising. The results indicate that looking at specific emotions may help to understand why some individuals are exercising regularly and others are not. As non-regular exercisers also anticipated to feel positive emotions and regular exercisers also anticipated to feel negative emotions in their next exercise session, future research should not only focus on the valence of future-oriented affect. Therefore, we encourage researchers to involve relevant emotion categories when examining the impact of future-oriented emotions on exercise behavior. In addition, the derived theoretical model of anticipated emotion categories may help to understand how specific anticipated emotions develop based on appraisal processes. This approach could be applied to practice during which coaches, teachers, and sport psychologists try to comprehend the exercise-related emotions of athletes, students, and exercisers. The role of anticipated emotions within the context of exercising should be investigated in future research considering the postulated cyclical process.

## Data Availability Statement

The raw data supporting the conclusions of this article will be made available for other researchers by request.

## Ethics Statement

The studies involving human participants were reviewed and approved by the Ethics Committee of the Karlsruhe Institute of Technology. The patients/participants provided their written informed consent to participate in this study.

## Author Contributions

KF conceptualized the study design and the interview manual under the supervision of DJ. HW provided methodological guidance. KF performed data interpretation with the assistance of SW, JF, and DJ. KF prepared and wrote the original draft in addition to conducting and transcribing interviews. SW, JF, HW, and DJ reviewed the original draft. All authors contributed to the article and approved the submitted version.

## Conflict of Interest

The authors declare that the research was conducted in the absence of any commercial or financial relationships that could be construed as a potential conflict of interest.

## Publisher’s Note

All claims expressed in this article are solely those of the authors and do not necessarily represent those of their affiliated organizations, or those of the publisher, the editors and the reviewers. Any product that may be evaluated in this article, or claim that may be made by its manufacturer, is not guaranteed or endorsed by the publisher.
