# Supplementary material for: Anticipatory and Anticipated Emotions in Regular and Non-regular Exercisers – A Qualitative Study
Source: Front Psychol. 2022 Jul 4;13:929380. doi: 10.3389/fpsyg.2022.929380 (PMC9302565; doi:10.3389/fpsyg.2022.929380)
Supplement: Supplementary file 1 [file Data_Sheet_1.pdf]

Supplementary Material

Interview manual

Created and compiled by Katharina Feil and Prof. Dr. Darko Jekauc (Karlsruhe Institute of Technology, Germany)

Information about the study and the interview:

First of all, I would like to thank you for participating in the study and am pleased that we could find a date to carry out the interview.

My name is Katharina Feil and I am conducting this study. I studied sports science with the profile physical activity and health at the KIT. My focus is on health psychology and the question of what motivates people to stay fit and healthy. This study is about finding out which emotions arise when thinking about future exercise sessions.

As a side note, I will not respond to your answers as often as I would in a normal conversation in order to prevent my judgment influencing the interview. This might feel a bit unusual at the beginning, but just take the chance to talk without any interruption. At the beginning, I would like to know what kind of sport or exercise you do and what your sports program currently looks like. Later on, the interview will be divided into two parts, but they might seem very similar to you. In the first part, you have to describe the feelings you have right now when you think about something. In the second part you have to imagine yourself in a situation and think about how you may feel in this situation. The difference is that, in the first part, you have to describe your current feelings and, in the second part, you have to reflect and describe the emotions that you expect to arise in the future. Do you have any questions about this explanation?

To facilitate the evaluation, I would like to record our conversations. Do you consent to the conversation being recorded? All information provided will of course be treated anonymously, confidentially and with the utmost care. Participation is voluntary and you have the option at any time to not answer a question or to terminate the interview. Do you have any questions about the process? Otherwise we would start the interview now.

Getting to know the participant:

- Ice-Breaker: In what kind of sport or exercise are you most involved in at the moment?
- How did you get involved?
- What does your sport or exercise program look like at the moment?
- How long have you been practicing in the way you are doing it at the moment?

Role of exercising in the participants’ life:

- What are the reasons for you exercising the way you do at the moment? Regarding the frequency, the place, the provider.
- How important is sport or exercising in your life?
- What are the reasons for why exercising should be part of your life?

Part 1: Anticipatory emotions

We begin now with the first part which regards your emotions at the moment. You probably remember that I mentioned this earlier.

- What do you feel right now when you think about your next sport or exercise session?
  - Can you describe this feeling in more detail?
  - What thoughts come to your mind spontaneously when you think about the next sport or exercise session?
  - What physical sensations can you feel?
  - What name would you give this emotion?
  - Can you describe a situation in the past from which this feeling might have developed/ in which this feeling arises?
  - Why do you think this feeling is related to the situation?
- Do any other feelings come up when you think about the next exercise session? If so:
  - Can you describe this feeling in more detail?
  - What thoughts come to your mind spontaneously when you think about the next sport or exercise session?
  - What physical sensations can you feel?
  - What name would you give this emotion?
  - Can you describe a situation in the past from which this feeling might have developed/ in which this feeling arises?
  - Why do you think this feeling is related to the situation?

Part 2: Anticipated emotions

We begin now with the second part, in which you are supposed to imagine what your next sports or exercise session will look like. If it helps you, you can close your eyes for a moment and think about all the things that will be part of your next sports or exercise session.

- What feelings are you expecting during the next sport or exercise session?
  - Can you describe this feeling in more detail?
  - What physical sensations can you feel during the sports or exercise session?
  - What name would you give this emotion?
  - Why do you think you will feel this way?
- Do you expect any other feelings in the next sports or exercise session? If yes:
  - Can you describe this feeling in more detail?
  - What physical sensations can you feel during the sports or exercise session?
  - What name would you give this emotion?
  - Why do you think you will feel this way?
- What feelings are you expecting immediately after the next sport or exercise session?
  - Can you describe this feeling in more detail?
  - What physical sensations can you feel during the sports or exercise session?
  - What name would you give this emotion?
  - Why do you think you will feel this way?
